# Changes in illness representations in patients with somatoform disorder after group-analysis therapy: Comparisons to psychoeducation program

**DOI:** 10.1192/j.eurpsy.2021.499

**Published:** 2021-08-13

**Authors:** I. Belokrylov, S. Semikov, E. Rasskazova, A. Tkhostov, V. Sadovnichaja

**Affiliations:** 1 Department Of Psychiatry And Medical Psychologi, Peoples Friendship University of Russia (RUDN University), Moscow, Russian Federation; 2 Clinical Psychology, Moscow State University, Moscow, Russian Federation; 3 Department Of Neuro- And Pathopsychology, Faculty Of Psychology, Lomonosov Moscow State University, Moscow, Russian Federation

**Keywords:** group analysis, somatoform disorders, illness representation

## Abstract

**Introduction:**

Psychological work with cognitive beliefs were shown to be beneficial for patients with somatoform disorders and unexplained somatic complaints (Liu et al., 2019). There is still a question of whether these results are specific or common for different kind of interventions including psychoanalytic psychotherapy (Kaplan, 2014).

**Objectives:**

The aim was to reveal dynamics of illness perception after group analysis psychotherapy comparing to psychoeducation in patients with somatoform disorders.

**Methods:**

100 patients with somatoform disorders were randomized to psychoeducation intervention (48 patients; 15 males and 33 females) and to the group analysis psychotherapy (52 patients; 15 males and 37 females). Before and after treatment they filled Screening for somatoforms symptoms (Rief, Hiller, 2003) and Illness Perception Questionnaire - Revised (Moss-Morris et al., 2002).

**Results:**

2 (Groups) × 2 (Time: Before / After) ANOVA with repeated measures revealed major effect of time with both groups demonstrated equal decrease in somatoform symptoms during treatment (F=101.42, p<.01, η²=.52). Patients from both groups after treatment appraised their illnesses as having shorter duration without cycles, less severe consequences on their lives, reported increase in treatment control, understanding of their illness and decrease in emotional reactions (F=7.13-30.62, p<.01, η²=.07-.24). In group analysis condition only patients demonstrated increased beliefs that psychological and risk factors could impact their illness (interaction: F=4.58-7.24, p<.05, η²=.05-.07).

**Conclusions:**

Patients with somatoform disorders almost equally benefitted from both psychoeducation and group analysis but group analysis psychotherapy led to better awareness of psychological and risk factors of their illness.

